# Effectiveness of Individual Psychoeducational Interventions for Caregivers of Stroke Patients: A Systematic Review and Meta-Analysis

**DOI:** 10.1007/s10880-025-10097-x

**Published:** 2025-10-15

**Authors:** Hesham Kelani, Hossam Tharwat Ali, Ahmed Naeem, Hazem Mohamed Salamah, Ali Ismail, Youmna Atef Younes, Ismail A. Ibrahim, Ahmed Fikry Mohamed, Abdelrahman Mady, Ahmed Abd Elazim, Mohammad El-Ghanem, Volodymyr Vulkanov, Diana Greene-Chandos, May Noor, David P. Lerner, Arthur D. Kay, Lisa R. Merlin, Priyank Khandelwal

**Affiliations:** 1 Department of Neurology, SUNY Downstate Health Sciences University at One Brooklyn Health, Brooklyn, USA; 2 Faculty of Medicine, South Valley University, Qena, Egypt; 3 Department of Neuroradiology, MD Anderson Cancer Center, University of Texas, Texas, USA; 4 Faculty of Medicine, Zagazig University, Zagazig, Egypt; 5 Faculty of Medicine, Lebanese University, Beirut, Lebanon; 6 Faculty of Medicine, Kaferelshiekh University, Kaferelshiekh, Egypt; 7 Faculty of Health Sciences, Fenerbahce University, Istanbul, Turkey; 8 Department of Neurology, University of South Dakota, Sanford Medical Center, Sioux Falls, USA; 9 Department of Clinical Science, University of Houston, HCA Houston-Northwest Medical Center, Houston, USA; 10 Department of neurology, Rutgers New Jersey School of Medicine, Newark, USA; 11 Department of Neurology, School of Medicine, Saint Louis University, St Louis, USA; 12 Department of Radiology, University of California, Los Angeles, USA; 13 Department of Neurology, Physiology, and Pharmacology, SUNY Downstate Health Sciences University, Brooklyn, USA; 14 Department of Neurosurgery, Rutgers New Jersey School of Medicine, Newark, USA

**Keywords:** Individual, Psychoeducation, Meta-analysis, Quality of life, Stroke, Caregivers

## Abstract

Stroke is a major cause of disability, and patients who suffer strokes have limited mobility and functional tasks, necessitating daily reliance on caregivers. However, caregivers of stroke patients often experience depression and anxiety, negatively impacting their mental health and reducing their quality of life. Psychoeducational interventions may be a solution to support the well-being of stroke caregivers. This study is performed to assess the overall effectiveness of individual psychoeducational interventions for caregivers of stroke patients. A thorough search of Scopus, PubMed, Web of Science, and Cochrane databases was performed for published studies in English up to June 2023. Clinical trials assessing the efficacy of psychoeducational interventions on quality of life, depression, or care burden among stroke caregivers compared to usual care were included. A total of 18 clinical trials, 16 randomized clinical trials (RCTs), and two non-RCTs, with a total of 2007 patients, were included. The study's pooled results revealed a significant increase in the quality of life in the group receiving psychoeducational interventions compared to the comparison group (SMD = 0.34, 95% CI 0.13–0.55, *p* value = 0.002), while no significant difference was found in terms of depression (SMD =  − 0.05, 95% CI  − 0.23 to 0.14, *p* value = 0.62) or caregiver burden (SMD =  − 0.61, 95% CI  − 1.65 to 0.44, *p* value = 0.25). Psychoeducation programs should be considered as a supportive intervention to improve quality of life in caregivers; however, their impact on depression and caregiver burden remains inconclusive. However, further studies with a larger sample size are needed to confirm the results.

**Supplementary Information:**

The online version contains supplementary material available at 10.1007/s10880-025-10097-x.

## Introduction

A stroke is defined as an acute focal neurological dysfunction, either due to ischemic or hemorrhagic origin, that persists for more than 24 h (Sacco et al., 2013; Shatri & Senst, 2018). Globally, stroke is a major cause of disability and the second leading cause of death with more than 15 million individuals having stroke annually (Katan & Luft, 2018; World Health Organization., 2020). Its incidence is rising with the rise of population aging and prolonged life span. Starting from the age of 25, both males and females face a lifetime risk of stroke of 25% (Katan & Luft, 2018). Being a life-threatening and potentially disabling condition, stroke causes a huge global burden (Dewey et al., 2002).

Living with disability constitutes numerous challenges for both survivors and caregivers who support them. Stroke patients were found to face difficulties related to limited mobility and inability to carry out basic functional tasks which made them depend on their caregivers in daily life (Kalavina, 2019). This can cause psychological, social, and physical burden on the caregivers (Schulz & Beach, 1999). The demanding nature of caregiving can significantly impact their quality of life, emotional well-being, and overall mental health (Scholte Op Reimer et al., 1998; Takai et al., 2009). Care burden is defined as the level of multifaceted strain experienced by a caregiver from caring for a family member for a long time (Liu et al., 2020; Scholte Op Reimer et al., 1998). Additionally, the previous studies showed a high prevalence of depression and anxiety symptoms among caregivers of stroke patients, which were 40.2% and 21.4%, respectively (Loh et al., 2017). Consequently, caregivers of stroke survivors are at an increased risk of developing mental health problems as well as reduced quality of life (McCullagh et al., 2005; Omar et al., 2021; Wan-Fei et al., 2017).

Previous studies investigated the impact of stroke on caregivers and explored interventions aimed at improving their well-being. To overcome these problems, interventions have been developed to support the well-being of stroke caregivers. These interventions include psychological, educational, and support groups provided to only caregivers or caregiver–survivor dyads (Hong et al., 2017; Panzeri et al., 2019). These interventions typically involve providing caregivers with information, guidance, and support to improve their understanding of stroke-related issues, develop coping strategies, and thus reduce stress and improve quality of life (Sarkhel et al., 2020). There is no consensus in the literature regarding the specific definition of quality of life (Chen, 2022; Estoque et al., 2019; Salvador-Carulla et al., 2014; Van Leeuwen et al., 2019); however, the World Health Organization (WHO) defines it as ‘an individual’s perception of their living situation, understood from a cultural and value system context, and in relation to the objectives, expectations, and standards of a given society’ (World Health Organization., n.d.).

While such interventions can be provided individually to the caregivers or the caregiver–survivor dyad, several reviews have attempted to examine the effectiveness of dyadic psychoeducational interventions for stroke survivors and caregivers (Cheng et al., 2014; Minshall et al., 2019; Mou et al., 2021; Pucciarelli et al., 2021). Although previous reviews have provided valuable insights into dyadic psychoeducational interventions, no review summarized the evidence about the effectiveness of psychoeducational interventions given individually to caregivers. Exploring the comparison of the effectiveness of dyadic and individual interventions will help tailor the most efficient method to standardize such interventions. Our review aims to address this gap in the literature pertaining to the effects of individual psychoeducational interventions for stroke caregivers concerning quality of life, depression, and burden of care.

## Methods:

### Protocol and Registration

The present systematic review and meta-analysis was carried out according to the Preferred Reporting Items for Systematic Reviews and Meta-analyses (PRISMA) statement (Liberati et al., 2009; Moher et al., 2009). The protocol was registered on the Prospero website (CRD42023493515), the international prospective register of systematic reviews, available at https://www.crd.york.ac.uk/prospero/display_record.php?RecordID=493515.

### Literature Search and Selection Criteria

We searched different databases such as Scopus, PubMed, Web of Science, and Cochrane for published studies in English up to June 2023. We constructed a thorough search string using relevant keywords (Appendix 1). The published reviews and reference lists of selected papers were also searched. Additionally, we searched through the ClinicalTrials.gov website up to June 2023.

We included clinical trials assessing the efficacy of individual psychoeducational interventions on quality of life, depression, or care burden among stroke caregivers. We included psychoeducational interventions intentionally given to caregivers only including information giving and discussion of relevant skills, guidance on problem-solving with stroke cases, counseling, social support, and/or behavior therapy. Exclusion criteria were review articles, theses, conference abstracts, editorials, commentaries, case reports, and articles written in languages other than English (Appendix 2).

### Selection/Screening of Studies

Two authors independently screened titles and abstracts of the citations retrieved by the literature search against the inclusion and exclusion criteria. We retrieved the full text of the potentially eligible records. Two different independent reviewers screened the full-text papers against the inclusion criteria, with the reconciliation of any differences conducted by a third independent reviewer.

### Data Extraction and Outcome Measures

Finally included studies underwent data extraction using specifically designed extraction forms. Two independent reviewers extracted data; a third independent reviewer resolved any differences. Extracted data included but was not limited to study methodology and design, participants' characteristics, and outcome measures. Efficacy outcome measures included changes in quality of life, depression level, or care burden of stroke caregivers.

### Assessment of Risk of Bias

The Cochrane risk-of-bias 2 tools (ROB2) were used for randomized and cluster randomized trials, whereas, for non-randomized studies of interventions, we used the Cochrane risk of bias in non-randomized studies—of interventions (ROBINS-I) tool. For each study, two authors independently assessed the risk of bias, and a third author resolved any differences.

### Data Analysis

All analyses were performed using RStudio version 2023.12.0 + 369 using the "meta" package. The standardized mean difference (SMD) with 95% confidence intervals (CI) was utilized to synthesize the pooled results from continuous outcomes. For precise estimation of efficacy, changes in mean scores between pre-test and post-test within comparable groups were employed to estimate treatment effects. Subgroup analysis was done based on the duration of follow-up and was categorized into three groups: immediate (< 1 month), short-term (1 month to < 6 months), and long-term (≥ 6 months) post-intervention (Mou et al., 2021). Given variations in intervention delivery modes, formats, sessions, and durations, random-effect models were employed for pooled analyses. Heterogeneity assessment involved visual inspection of forest plots, Cochrane's *Q* (*X*^2^ test), and *I*^2^ statistics. *I*^2^ cut-offs of < 25% (no heterogeneity), 25–50% (low heterogeneity), 50–75% (moderate heterogeneity), and > 75% (high heterogeneity) were employed (Pigott, 2012). Significance was determined by an *X*^2^ test for *Q* (*p* value ≤ 0.05). In case of significant marked heterogeneity, the Cochrane leave-one-out method was applied, excluding one study from the analysis.

## Results

### Search Results

**Fig. 1:**
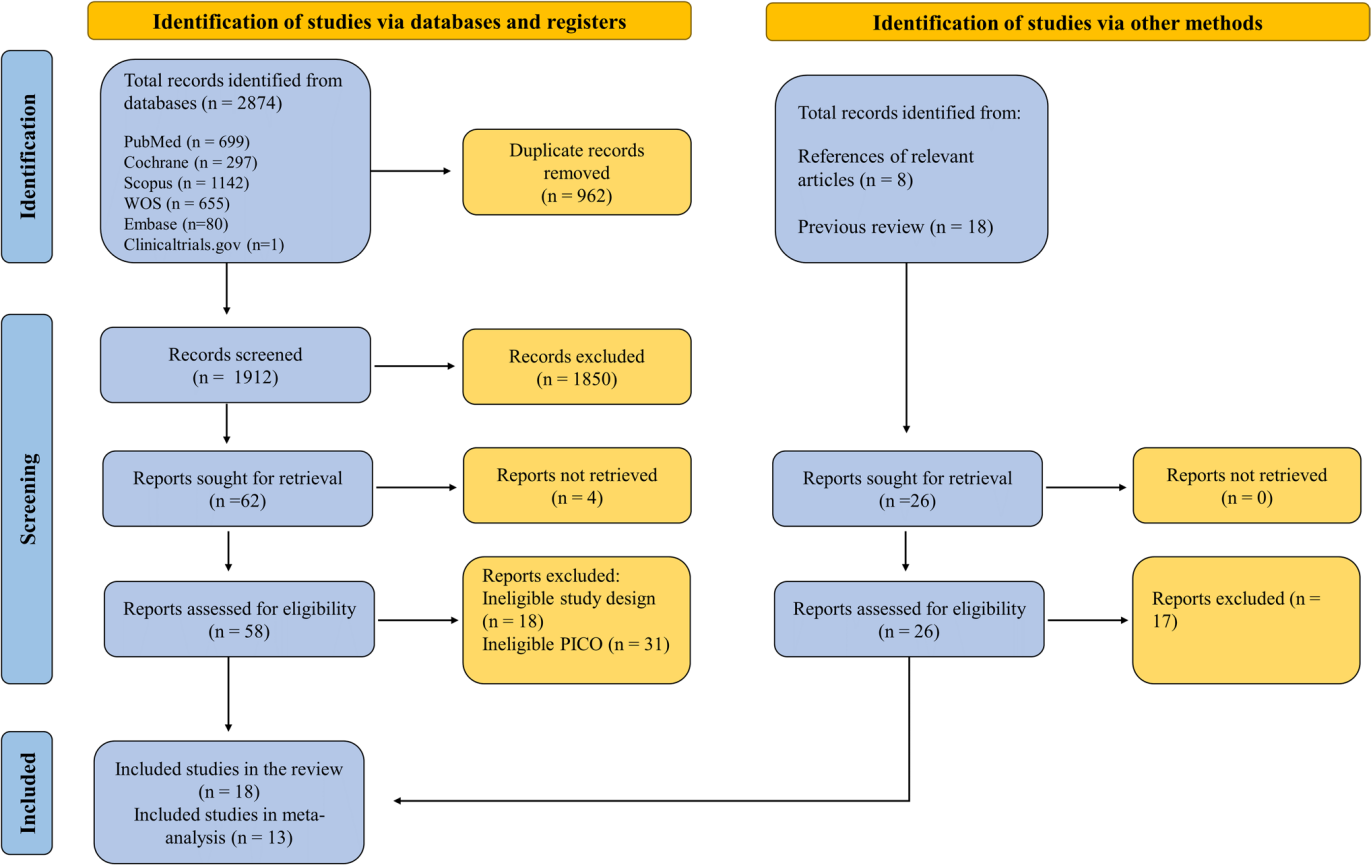
A PRISMA flow diagram shows the screening process

In total, searching databases yielded 2874 records. Out of these, 962 duplicate records were detected and eliminated, resulting in a remaining count of 1912 records to be examined. By reviewing titles and abstracts, we excluded 1850 records that did not meet our predefined eligibility criteria. Four reports could not be retrieved. A meticulous evaluation was conducted of the remaining 58 full-text articles with the exclusion of 49 articles. In addition to the final nine articles, nine articles were detected and deemed eligible from previous reviews and references, yielding a total of 18 included in the review (Fig. 1).

### Characteristics of the Included Studies and Patients

Of the 18 included trials, 15 were randomized controlled trials (RCTs) (Farahani et al., 2021; Bani Ardalan et al., 2022; Bierhals et al., 2023; Day et al., 2021; Draper et al., 2007; Elsheikh et al., 2022; Goudarzian et al., 2018; Grant et al., 2002; Hartke & King, 2003; Hekmatpou et al., 2019; İnci & Temel, 2016; King et al., 2012; Larson et al., 2005; Pfeiffer et al., 2014; Van Den Heuvel et al., 2002), one cluster randomized trial (Rodríguez-Gonzalo et al., 2015), and two non-randomized trials (Araújo et al., 2018; Gräsel et al., 2005) with a total population size of 2007 patients. All studies included adult patients with variable sample sizes from 39 to 255 participants. The study duration varied from two weeks to one year. The interventions employed in the studies focused on one or both of the educational or psychological elements for only caregivers. The educational part involves providing relevant information on stroke management plans, feeding methods and instructions, mobility, transferring information, and/or skill training. Psychological support was provided in the form of support teams, behavioral therapy, stress management, and/or emotional self-care. A detailed description of interventions is provided in Table 1. It is noteworthy that such interventions have been implemented using in-person sessions, online sessions, or telephone-based consultations. The summary of study characteristics is shown in Tables 1 and 2**.** The majority of caregivers were females with mean age ranging from 43.41 ± 11.25 to 69.74 ± 5.39 years. Appendix 3 contains the baseline characteristics of the participants.

**Table 1:** Summary of Characteristics of the Included Studies

Reference/study	Study design/phase	Country/region	Study duration	Total sample size (intervention/comparison)	Intervention	Comparison
Van Den Heuvel et al. (2002)	RCT	Netherlands	7 months	212 (170/42)	∙Content: Structured support program including emotional expression, information-sharing, and active coping strategies ∙Method of delivery: Group sessions or individual home visits ∙Provider: Health education nurse	Usual care
Grant et al. (2002)	RCT	US	12 weeks	74	∙Content: Social problem-solving intervention (SPTPs) ∙Method of delivery: Initial home visit followed by structured telephone-based sessions ∙Provider: Trained nurse	∙Sham intervention Comparison group: usual care
Hartke & King. (2003)	RCT	US	6 months	88 (43/45)	∙Content: Psychoeducation and behavioral training, supplemented with a relaxation audiotape and a stress management publication ∙Method of delivery: Telephone-based ∙Provider: Not specified	Usual care + manual stress management publication
Grasel et al. (2005)	Controlled clinical trial (Non-randomized)	Germany	6 months	62 (33/29)	∙Content: Psychoeducation and skill-building transitional support program ∙Method of delivery: In-person home counseling and telephone-based follow-ups ∙Provider: Not specified	Usual care
Larson et al. (2005)	RCT	Sweden	12 months	97 (47/50)	∙Content: Structured support and education program focused on stroke-related caregiving challenges ∙Method of delivery: In-person ∙Provider: Stroke specialist nurse	Regular information during the patient’s hospitalization and at the discharge
Draper et al. (2007)	Randomized wait-list controlled trial	Australia	8 months	39 (19/20)	∙Content: SHARE intervention, including caregiver education, psychological support, and skill training	Usual care. They received the treatment program after a delay of three months
King et al. (2012)	RCT	NR	One year	255 (136/119)	∙Content: Caregiver problem-solving intervention (CPSI) ∙Method of delivery: In-person and telephone-based counseling ∙Provider: Nurse practitioners and psychology doctoral students	Usual care (Wait-list control)
Pfeiffer et al. (2014)	RCT	Germany	12 months	122 (60/62)	∙Content: Problem-solving intervention (PSI), including two home visits and 18 telephone sessions ∙Method of delivery: including two home visits and 18 telephone sessions through 3-month intensive phase and 9-month maintenance period ∙Provider: Clinical psychologists	Comparison groups received monthly information letters in addition to usual care
Rodríguez-Gonzalo et al. (2015)	Cluster randomized trial	Spain	One month	151 (78/73)	∙Content: Intensive education on feeding, hygiene, mobility, and emotional self-care ∙Method of delivery: Structured in-person training sessions ∙Provider: Not specified	A single 2-h session, taught weekly, on generic life and aging processes
Inci et al. (2016)	RCT	Turkey	6 months	80 (40/40)	∙Content: Structured support program combining educational sessions and social support meetings	∙Routine home care
Goudarzian et al. (2018)	RCT	Iran	3 months	152 (76/76)	∙Content: Educational telenursing intervention ∙Method of delivery: Telephone-based sessions ∙Provider: Nurse	Usual care
Araujo et al. (2018)	Quasi-experimental design (Non-randomized)	Portugal	3 months	174 (85/89)	∙Content: InCARE program providing home-based training on mobility, personal care, and use of assistive devices ∙Method of delivery: In-person home sessions ∙Provider: Nurse	Only standard and medical caregiving education by nurses in healthcare units
Hekmatpou et al. (2019)	RCT	Iran	One month	100 (50/50)	∙Content: Educational counseling on stroke caregiving ∙Method of delivery: In-person hospital-based training with telephone support and an instructional booklet ∙Provider: student of Master of Science in Nursing	Routine training
Day et al. (2021)	RCT	Brazil	One year	48 (24/24)	∙Content: SHARE caregiving education program emphasizing reflective thinking and collaborative problem-solving ∙Method of delivery: In-person home sessions ∙Provider: Trained nurses	Usual care during hospitalization and discharge
Farahani et al. (2021)	RCT	Iran	2 weeks	116 (58/58)	∙Content: Comprehensive supportive home care program integrating education, behavioral training, and skill development ∙Method of delivery: In-person home visits, telephone consultations, and peer support ∙Provider: PhD candidate in nursing	Routine hospital education program about CVA and caring for patients with CVA
Ardalan et al. (2022)	RCT	Iran	12 weeks	79 (39/40)	∙Content: 12-week educational program ∙Method of delivery: WhatsApp-based learning with regular telephone follow-ups ∙Provider: Not specified	Usual care
Elsheikh et al. (2022)	RCT	Egypt	6 months	110 (55/55)	∙Content: Tailored multidimensional intervention incorporating psychoeducation, skill-building, and peer support ∙Method of delivery: In-person home visits and telephone calls ∙Provider: Experienced nurses	Simple educational instructions at a single visit
Bierhals et al. (2023)	RCT	Brazil	One year	48 (24/24)	∙Content: SHARE caregiving education program focusing on reflective thinking and shared problem-solving ∙Method of delivery: In-person home sessions ∙Provider: Trained nurses	Usual care during hospitalization and discharge

*RCT* randomized controlled trials, *CVA* cerebrovascular accidents, *SPTPs* social problem-solving intervention, *CPSI* caregiver problem-solving intervention, *PSI* problem-solving intervention

**Table 2:** Endpoints and outcomes of the included studies

Reference/study	Study outcome measures (scale or tool)	Data collection time points	Outcome findings
Van Den Heuvel et al. (2002)	∙Confidence in knowledge ∙Coping strategies (short version of the Utrecht coping list) ∙Physical well-being (SF-36) ∙Social support and satisfaction with social support (Adapted versions of the Social Support List-Interaction and the Social Support List-Discrepancy) ∙Assertiveness	∙baseline ∙One month ∙7 months	In the intervention group, confidence in knowledge and coping strategies improved over time. Social support remained stable in the intervention group but declined in the comparison group. However, no significant differences were observed between group support and home visits
Grant et al. (2002)	∙General Health (SF-36) ∙Social Problem-Solving Abilities (The Social Problem-Solving Inventory) ∙Satisfaction With Health Care (The Client Satisfaction Questionnaire) ∙Depression (CES-D scale) ∙Caregiver Preparedness ∙Caregiving Burden	∙Baseline ∙12 weeks	There were significant improvements in general health, social problem-solving abilities, and caregiver preparedness in the intervention group. However, no significant impact was noted on caregiver burden. Satisfaction with healthcare remained stable in the intervention group but declined in the comparison group
Hartke & King. (2003)	∙Depression (CES-D scale) ∙Caregiver’s burden (BI) ∙Loneliness (UCLA Loneliness Scale) ∙Stress (PPI) ∙Caregiver competence	∙Baseline ∙Immediately after the intervention ∙6 months	Caregivers in the intervention group experienced increased competence, while the comparison group showed a rise in caregiver burden
Grasel et al. (2005)	∙Depression (Zerssen Depression Scale) ∙Somatic complaints (GSL-24) ∙Caregiver’s burden (BSFC) ∙Patient’s function and dependence (Barthel index + FIM)^a^ ∙Patient’s quality of life (SF-36)^a^	∙Baseline ∙6 months	The intensified transition did not lead to significant changes in the functional status of the patients or carer-based measures
Larson et al. (2005)	∙Quality of life (visual analog scale) ∙The health status (the graded visual analog scale part of the EuroQoL-instrument) ∙Life situation (LISS questionnaire) ∙Well-being (Bradley’s well-being questionnaire)	∙Baseline ∙6 months ∙12 months	No significant differences were found between the intervention and comparison groups. However, participants who attended more sessions in the intervention group experienced improved well-being and reduced negative emotions
Draper et al. (2007)	∙Psychological distress (GHQ) ∙Caregiver burden (RSS) ∙Communication (ComA and ComB)	∙Baseline ∙One month ∙4 months	Intervention resulted in a short-term reduction in caregiver stress, but long-term benefits required continued participation in the program.
King et al. (2012)	∙Depression (CES–D) ∙Perception of Life Change (BCOS) ∙Caregiver Preparedness (The Preparedness for Caregiving Scale) ∙Anxiety (the Tension-Anxiety 5-item subscale of the Profile of Moods Scale) ∙Family functioning (the General Functioning Scale of the McMaster FAD)	∙Baseline ∙3 to 4 months ∙6 months ∙12 months	Initial improvements in depression and life changes among caregivers in the intervention group were observed, though these effects diminished over time.
Pfeiffer et al. (2014)	∙Depression (CES–D) ∙Caregiver Competence (SCQ) ∙Social problem-solving abilities (SPSI–R:S) ∙Physical complaints (GBB–24) ∙Satisfaction with leisure time (LTS)	∙Baseline ∙3 months ∙12 months	Intervention resulted in a reduction in depressive symptoms at three months, which was maintained at twelve months. However, no significant improvement in problem-solving skills was observed
Rodríguez-Gonzalo et al. (2015)	∙Quality of life (SF-12) ∙Caregivers’ burden (Zarit's test) ∙Caregiving knowledge	∙Baseline ∙Immediately after the intervention ∙One month	Participants' quality of life and burden did not improve on the intervention nor did the overall caregiving knowledge Only mobility and hygiene knowledge improved significantly
Inci et al. (2016)	∙Resilience, adjustment, and adaptability (FIRA-G)	∙Baseline ∙One month ∙6 months	Intervention resulted in significant improvements in social support and family coping mechanisms, with a reduction in family distress over time
Goudarzian et al. (2018)	∙Depression (BDI) ∙Anxiety (BAI)	∙Baseline ∙3 months	There was a significant difference in anxiety scores of both groups after intervention. However, the difference in depression scores was not significant
Araujo et al. (2018	∙Self-care skills (ECPICID-AVC) ∙Burden (QASCI) ∙Global Health Condition (SF-36)	∙One month ∙3 months	The intervention produced significantly better results regarding practical skills as well as lower burden levels and a better general mental health condition when compared with the comparison group 1 and 3 months after the intervention
Hekmatpou et al. (2019)	∙Care burden (Zarit burden scale) ∙Quality of life (SF-36)	∙Baseline ∙One month	Patient care education reduced the burden of care and improved the quality of life of the caregivers of stroke patients
Day et al. (2021)	∙Caregiver burden (CBS)	∙Baseline ∙2 months ∙1 year	The intervention had a statistically significant effect on the caregivers’ burden with respect to isolation and emotional involvement domains
Farahani et al. (2021)	∙Caregiver burden (CBI)	∙Baseline ∙2 weeks	The intervention reduced the burden significantly, while the burden increased after two weeks in the comparison group
Ardalan et al. (2022)	∙Care burden (ZBI)	∙Baseline ∙12 weeks	Education & telephone follow-up for 12 weeks caused a significant reduction in the care burden of the family caregivers
Elsheikh et al. (2022)	∙The care burden (the short version of the ZBI) ∙Quality of life (The WHOQOL-BREF)	∙Baseline ∙3 months ∙6 months	Participants in the IG did not experience an improvement in the main outcomes
Bierhals et al. (2023)	∙Quality of life (WHOQOL-BREF + WHOQOL-OLD) ∙Functional capacity of the survivors (FIM)^a^	∙Baseline ∙2 months ∙1 year	The intervention exerted a statistically significant effect on the quality of life of family caregivers with respect to social relationships and autonomy

^a^Outcomes on stroke survivors

### Risk of Bias

Of the randomized trials, nine studies were decided as “having some concerns,” while six were deemed to be of “high risk.” Most studies had a low risk of bias in the domains of the randomization process and deviation from intended outcomes. While nine studies had a low risk of bias in the domain of missing outcome data, most studies did not explain methods to manage missing data. Some of the methods mentioned to manage missing data are the hierarchical linear model (Gräsel et al., 2005), the last observation carried forward method (Bierhals et al., 2023; Elsheikh et al., 2022), and the maximum-likelihood multiple imputation (Pfeiffer et al., 2014). In the domain of the selection of reported results, 12 studies had some concerns. For the two non-randomized trials, one study had a critical level of risk of bias, while the other yielded “No information.” The details of the risk-of-bias results of all studies are shown in Appendix 4 and Appendix 5.

### Meta-Analysis

#### Caregivers’ Quality of Life

**Fig. 2:**
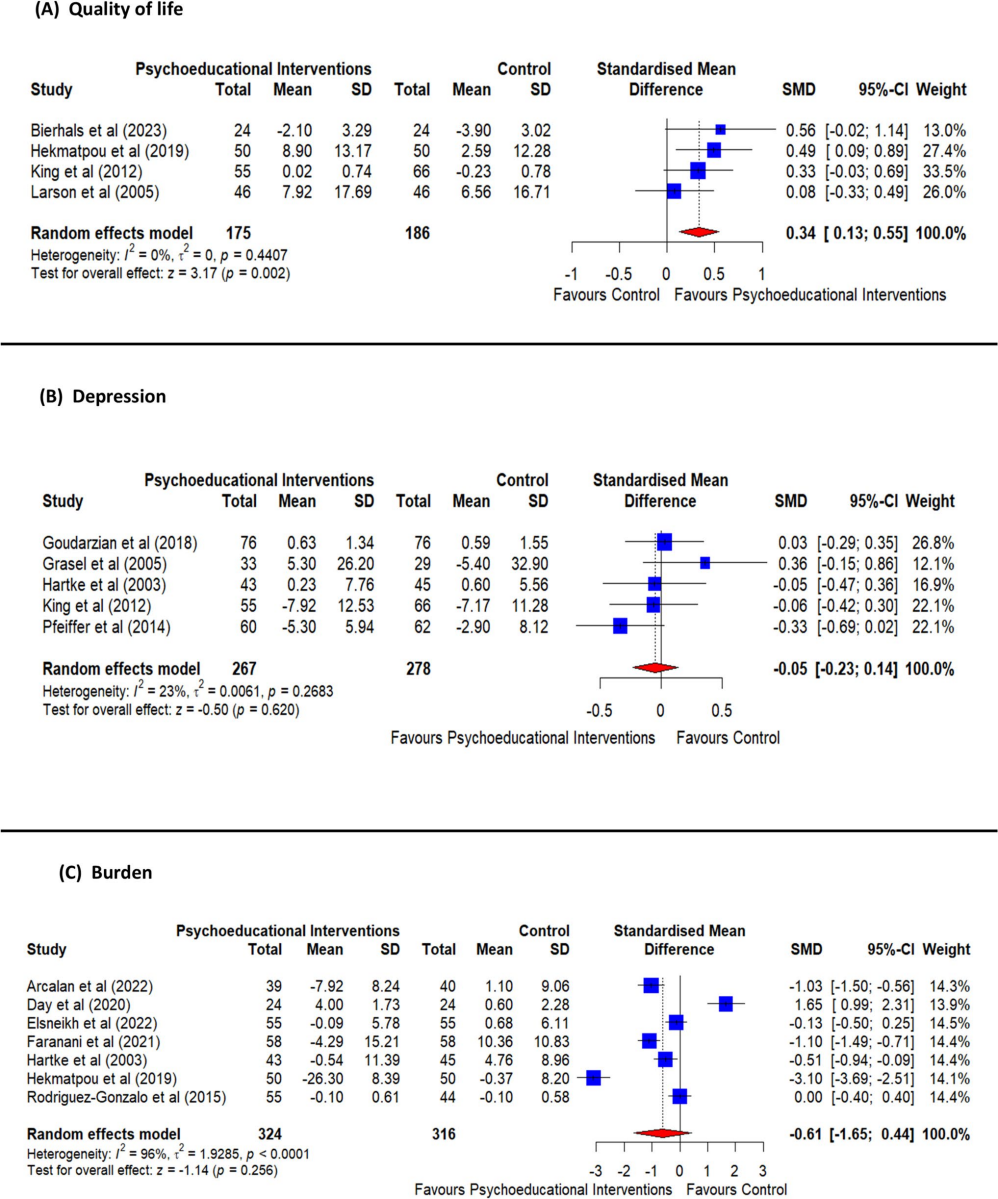
Forest plots show the effects of individual psychoeducational intervention on (a) the quality of life of stroke caregivers, (b) depression among caregivers of stroke patients, and (c) family caregivers’ burden

Figure 2a shows the pooled results of the four included studies examining the effects of individual psychoeducational intervention on the quality of life of stroke caregivers(Bierhals et al., 2023; Hartke & King, 2003; Hekmatpou et al., 2019; Larson et al., 2005). The psychoeducational intervention significantly raised the quality of life of stroke caregivers (SMD = 0.34, 95% CI 0.13 to 0.55, *p* value = 0.002). The pooled analysis was homogeneous (*I*^2^ = 0%, *p* value = 0.44).

Concerning subgroup analysis, the short-term results did not show a significant difference in the quality of life of stroke caregivers (SMD =  − 0.18, 95% CI  − 1.49 to 1.13, *p* value = 0.78). However, this intervention showed a significant increase in the quality of life of caregivers on a long-term level (SMD = 0.28, 95% CI 0.03 to 0.52, *p* value = 0.025). The heterogeneity of the short-term subgroup analysis was high (I^2^ = 94%, *p* value < 0.00001), while that of the long-term level was homogeneous (*I*^2^ = 0%, *p* value = 0.38) (Figure S1).

#### Caregivers’ Depression Level

Five included articles assessed the effect of individual psychoeducational intervention on depression among caregivers of stroke patients (Goudarzian et al., 2018; Gräsel et al., 2005; Hartke & King, 2003; King et al., 2012). The results showed a non-significant difference (SMD =  − 0.05, 95% CI  − 0.23 to 0.14, *p* value = 0.62). The pooled analysis was homogeneous (*I*^2^ = 23%, *p* value = 0.27) (Fig. 2b). Excluding the non-randomized clinical trial, Grasel et al. (2005), did not cause any significant difference (SMD =  − 0.10, 95% CI − 0.28 to 0.08, *p* value = 0.27). The pooled analysis was homogeneous (*I*^2^ = 0%, *p* value = 0.50) (Figure S2).

Regarding subgroup analysis, the results showed a non-significant difference at the short-term level between intervention and comparison groups (SMD =  − 0.25, 95% CI  − 0.54 to 0.04, *p* value = 0.10). Similarly, at the long-term level, the intervention group did not have a significant difference over the comparison group (SMD =  − 0.06, 95% CI  − 0.31 to 0.19, *p* value = 0.63). The pooled results were homogeneous for short-term (*I*^2^ = 54%, *p* value = 0.11) and long-term subgroups (*I*^2^ = 39%, *p* value = 0.18) (Figure S3).

#### Burden

Figure 2c shows the pooled results of seven studies assessing the effect of individual psychoeducational intervention on family caregivers’ burden (Farahani et al., 2021; Bani Ardalan et al., 2022; Day et al., 2021; Elsheikh et al., 2022; Hartke & King, 2003; Hekmatpou et al., 2019; Rodríguez-Gonzalo et al., 2015). The results showed a non-significant difference (SMD =  − 0.61, 95% CI  − 1.65 to 0.44, *p* value = 0.25). The pooled analysis was heterogeneous (*I*^2^ = 96%, *p* value < 0.0001). Sensitivity analysis did not resolve the heterogeneity (Figure S4).

Regarding subgroup analysis, immediately after the intervention, the caregiver burden was not reduced significantly after the intervention (SMD =  − 0.55, 95% CI  − 1.63 to 0.54, *p* value = 0.32). Similar results, no significant difference in caregivers’ burden, were found at the short-term level (SMD =  − 0.80, 95% CI  − 1.98 to 0.39, *p* value = 0.18), and the long-term level post-intervention (SMD = 0.31, 95% CI  − 0.97 to 1.60, *p* value = 0.63) (Figure S5). The pooled results were heterogeneous for all the subgroups.

## Discussion

Stroke is considered a family disease and the disability not only affects the patient but also affects the family members (Walsh, 1982). Providing care to a stroke survivor can impose significant physical, psychological, and emotional demands on the caregiver (Mou et al., 2021). Supporting the needs of stroke caregivers in the right format, time, and place is crucial for their and survivors’ lives. There have been many modalities and approaches for implementing supporting interventions for caregivers (Cheng et al., 2014). Interventions can be psychosocial, educational, or a combination provided to only caregivers, which is known as individual psychoeducation, or caregiver–survivor dyads. Many clinical trials, systematic reviews, and meta-analyses evaluated the efficacy of dyadic psychosocial and educational interventions in improving both caregivers’ and survivors’ psychosocial aspects (Cheng et al., 2014; Minshall et al., 2019; Mou et al., 2021; Pucciarelli et al., 2021). Dyadic educational interventions were found to induce a decrease in depression levels in caregivers (Pucciarelli et al., 2021). Similarly, psychosocial interventions provided for caregiver–survivor dyads caused improvement in quality of life and depression status in caregivers. Psychosocial interventions for caregivers only had a small effect on depression levels (Minshall et al., 2019). As for dyadic psychoeducational interventions, long-term improvement was noticed in quality of life among caregivers as well as an immediate reduction in care burden (Mou et al., 2021).

Numerous clinical studies have explored the effectiveness of such interventions focusing on individual caregiver support (Araújo et al., 2018; Farahani et al., 2021; Bani Ardalan et al., 2022; Bierhals et al., 2023; Day et al., 2021; Draper et al., 2007; Elsheikh et al., 2022; Goudarzian et al., 2018; Grant et al., 2002; Gräsel et al., 2005; Hartke & King, 2003; Hekmatpou et al., 2019; İnci & Temel, 2016; King et al., 2012; Larson et al., 2005; Pfeiffer et al., 2014; Rodríguez-Gonzalo et al., 2015; Van Den Heuvel et al., 2002). The present article comprises a comprehensive review of the evidence supporting the effectiveness of individual psychoeducational interventions on the psychosocial health outcomes of caregivers of stroke survivors. To the best of our knowledge, this is the first systematic review and meta-analysis of all approaches to caregiver-focused psychoeducational interventions in stroke care. The results suggest that individual psychoeducational interventions could elicit significant changes in caregivers’ quality of life. However, no significant effect was noticed on care burden as well as depression levels. It is noteworthy that the pooled analysis revealed that individual psychoeducation has significant effects on the caregivers’ quality of life, which is more evident with the longer duration of the intervention.

Depression is not uncommon among caregivers of stroke patients with challenging lifestyles and possible disabilities (Loh et al., 2017). The present analysis did not reveal any benefit of psychoeducational interventions in short-term, long-term, or overall assessment. This observation may be explained by the fact that depression is a complex disorder and multi-factorial in its origin including neurobiological (Dean & Keshavan, 2017), genetic (Saveanu & Nemeroff, 2012), environmental (Bosch et al., 2021), and immunological (Sarno et al., 2021) factors. Additionally, many different assessment scales were used for depression in the studies(Goudarzian et al., 2018; Grant et al., 2002; Hartke & King, 2003; King et al., 2012; Pfeiffer et al., 2014).

Caring for patients in daily life is generally challenging and dealing with stroke patients especially those with disabilities causes a huge burden on caregivers and major changes in their lives. This may be attributed to social factors, such as unemployment and violence, rather than the demands and implications of care. Therefore, even with education the caregiver may reduce the care burden, but social factors may have stronger effects. A recent review found strong evidence supporting the effectiveness of problem-solving combined with stroke education, as well as one-on-one caregiver education and support interventions for caregivers of people post-stroke (Mack & Hildebrand, 2023). Nevertheless, by pooling of largest number of studies in our meta-analysis, we found significant differences favoring the individual intervention group only after excluding Day et al., 2021 (Day et al., 2021). Notably, marked heterogeneity was detected even after the removal of outlier studies suggesting remarkable differences in patients’ characteristics, intervention approaches, and content, as well as assessment scales among the different studies.

### Strengths and Limitations

This systematic review is the first to assess the effectiveness of caregiver-focused individual psychoeducational interventions on their quality of life, care burden, and depression levels using a rigorous protocol. Furthermore, the results for all outcomes were derived from randomized clinical trials, except for the depression outcome. However, we conducted a sensitivity analysis by excluding the non-randomized clinical trial and reported the revised results. The findings remained consistent, with no significant differences compared to when the non-randomized trial was included.

However, there are some limitations. All the included studies in this review were relatively short-term, with follow-up periods of less than or equal to one year. Furthermore, there was marked heterogeneity among the studies. We tried to reduce the heterogeneity using the leave-one-out technique which could not resolve the heterogeneity. Notably, the studies varied in terms of intervention approaches, duration, and detailed content, and although all aimed to compare the intervention to the usual care, there is possible variability in comparisons among studies. Furthermore, regarding outcomes, the studies showed wide variability in their outcome measures reflecting that most studies were not designed solely to manage depression, burden, or quality of life which could impact the trajectory of change of each outcome.

It is noteworthy that most studies had some concerns or high risk of bias, especially in the domain related to outcome and results reporting. Publication bias is a common limitation of all systematic reviews, and it is difficult to quantify its impact on the results of this review (Higgins et al., 2019). It is noteworthy to state that in research involving caregivers and psychosocial elements, bias may exist as caregivers participate in such studies represent a certain group of those that are more motivated to comply with such interventions and caregiving practices, excluding those experiencing greater caregiving burdens and depression, making the outcomes not fully reflect the effectiveness of psychosocial interventions in the broader, more diverse caregiver population dealing with real-life challenges.

Despite these limitations, this systematic review provides a comprehensive overview of the existing evidence on individual psychoeducational interventions for stroke caregivers' support. The review also highlights the need for more long-term studies and studies that address the difference in intervention duration and approaches.

### Implications

Overall, it was shown that caregiver-focused individual psychoeducational interventions can be an effective option in addressing the changes in caregivers’ quality of life. Nevertheless, more studies with larger and representative samples, and more importantly, proper sampling methods are prompted. To attribute the effects of interventions to the duration, randomization should be pre-specified at the point of study duration not only the study intervention. Future studies could benefit from developing similar protocols in assessments of the trajectory of changes in the outcomes. Unifying study protocols especially with interventions, and standardized usual care (comparison) is also important to limit the heterogeneity that could be observed across studies. Although we used the mean change (between pre-test and post-test) in outcome measurement, unifying the study objectives and baseline level of participants in each outcome is recommended in future studies.

Due to the lack of significant changes in burden and depression, we recommend future studies focus on developing and evaluating interventions specifically tailored to address burden and depression among stroke caregivers. This could involve more personalized or intensive psychological support. It is important to study the effect of psychological and educational interventions separately for better understanding.

## Conclusions

In conclusion, this systematic review and meta-analysis has provided valuable insights into the effectiveness of psychoeducational interventions in improving some psychosocial aspects of stroke caregivers. Our findings indicate a possible benefit of such interventions in improving the caregivers’ quality of life. Further studies are needed to confirm and explain the findings regarding depression and care burden.

## Supplementary Information

Below is the link to the electronic supplementary material.

Supplementary file1 (DOCX 256 KB)

## Data Availability

Data are provided within the manuscript or supplementary information files.
