# Supplementary material for: Effectiveness of Individual Psychoeducational Interventions for Caregivers of Stroke Patients: A Systematic Review and Meta-Analysis
Source: J Clin Psychol Med Settings. 2025 Oct 15;33(1):158–73. doi: 10.1007/s10880-025-10097-x (PMC13035896; doi:10.1007/s10880-025-10097-x)
Supplement: Supplementary file 1 — Supplementary file1 (DOCX 256 KB) [file 10880_2025_10097_MOESM1_ESM.docx]

**Unveiling The Effect of Caregiver-Focused Individualized Psychoeducational Interventions: A Systematic Review and Meta-Analysis**

**Appendix Files**

**Appendix 1**

**The search strategy used through the databases**

(Stroke[MeSH] OR Stroke OR “Cerebrovascular Accident” OR Strokes OR “Cerebrovascular Accident” OR CVA OR CVAs OR “Cerebrovascular Apoplexy” OR “brain vascular accident” OR “brain vascular accidents” OR Infarction OR Infarctions OR Infarct OR Infarcts OR “Cerebral stroke” OR “cerebral strokes”))

AND

(Caregiver*[MeSH] OR Family OR families OR spouse OR spouses OR caregiver OR caregivers OR “care givers” OR “care giver” OR carers OR carer OR Supporter OR Guardian OR Custodian OR Warden OR Sitter OR Nurser OR Companion)

AND

(“Quality of life”[MeSH] OR “Quality of life” OR “Life Quality” OR “Health Related Quality of Life” OR HRQOL OR HRQL OR depression OR burden OR anxiety OR physical OR functional OR mental OR psychological OR psychosocial OR dependency OR stress OR distress OR wellbeing OR coping OR emotional)

**Appendix 2**

**Inclusion and exclusion criteria of the studies.**

**PICOS:**

- Population: Caregivers of stroke patients
- Intervention: Individual psycho-educational interventions
- Control: Sham procedure or usual care
- Outcome:
- Caregivers’ quality of life or overall health
- Caregivers’ depression
- Care burden
- Study Design: Clinical trials

**Inclusion criteria:**

Trials with PICOS as mentioned earlier will be considered for inclusion.

**Exclusion criteria:**

- Review articles, theses, conference abstracts, editorials, commentaries, and case reports.

- Articles written in languages other than English.

- Studies with unreliable data for extraction.

**Appendix 3**

**Demographic characteristics of the participants**

| **Reference / Study** | **Groups** | **Caregivers' Characteristics** | | **Stroke patients'/survivors' characteristics** | |
| --- | --- | --- | --- | --- | --- |
|  |  | **Age (years)** * | **Female (%)** | **Age (years)** * | **Female (%)** |
| **Van den heavily et al, 2002** | **Intervention** | NR | 73.5 | NR | 26.5 |
|  | **Control** | 60.8 | 71.4 | NR | 28.6 |
| **Grant et al, 2002** | **Intervention** | NR | NR | NR | NR |
|  | **Control** | NR | NR | NR | NR |
| **Hartke et al, 2003** | **Intervention** | 69.74 (5.39) | 74 | NR | NR |
|  | **Control** | 69.69 (6.59) | 78 | NR | NR |
| **Grasel et al, 2005** | **Intervention** | 59.7 (9.8) | 76 | 71.4 (8.2) | 39 |
|  | **Control** | 59.4 (9.5) | 72 | 74.4 (7.8) | 28 |
| **Larson et al, 2005** | **Intervention** | 68.16 (10.42) | 76 | 71.66 (9.91) | NR |
|  | **Control** | 66.66 (9.82) | 84 | 69.94 (10.36) | NR |
| **Draper et al, 2007** | **Intervention** | 64.05 (11.5) | NR | 69.21 (9.61) | NR |
|  | **Control** | 59.95 (14.88) | NR | 67.90 (10.87) | NR |
| **King et al, 2012** | **Intervention** | 54.5 (15.1) | 76.5 | 61.2 (14.6) | 42.6 |
|  | **Control** | 54.6 (13.3) | 80.7 | 61.5 (14.7) | 38.7 |
| **Pfeiffer et al, 2014** | **Intervention** | 66.7 (9.9) | 76.7 | 73.4 (7.5) | 31.7 |
|  | **Control** | 65.6 (10.1) | 79 | 72.9 (7.5) | 30.6 |
| **Rodríguez-Gonzalo et al, 2015** | **Intervention** | 54.8 (15.1) | 80 | 7.5 (70.5 - 87.25) **#** | 58 |
|  | **Control** | 52.7 (15.5) | 81 | 82 (71–86) | 48 |
| **Inci et al, 2016** | **Intervention** | NR | 100 | NR | NR |
|  | **Control** | NR | 100 | NR | NR |
| **Goudarzian et al, 2018** | **Intervention** | 49.04 (14.96) | NR | 66.96 (11.81) | NR |
|  | **Control** | 49.48 (15.05) | NR | 70.07 (10.05) | NR |
| **Araujo et al, 2018** | **Intervention** | 56 (11.2) | 89.9 | NR | NR |
|  | **Control** | 54.3 (11.5) | 88.2 | NR | NR |
| **Hekmatpou D et al, 2019** | **Intervention** | 48.52 | 72 | NR | NR |
|  | **Control** | 45.14 | 54 | NR | NR |
| **Day et al, 2020** | **Intervention** | 53.38 (11.91) | 95.8 | NR | NR |
|  | **Control** | 53.54 (14.05) | 79.2 | NR | NR |
| **Farahani et al, 2021** | **Intervention** | 43.98 (13.8) | 65.5 | 68.5 (13.16) | 44.8 |
|  | **Control** | 43.41 (11.25) | 77.6 | 64.65 (12.2) | 46.6 |
| **Ardalan et al, 2022** | **Intervention** | NR | NR | NR | NR |
|  | **Control** | NR | NR | NR | NR |
| **Elsheikh et al, 2022** | **Intervention** | 35 (25–55) **#** | 76.4 | 65.75 (10.34) | 18.2 |
|  | **Control** | 35 (25–57) **#** | 72.7 | 65.58 (9.94) | 25.5 |
| **Bierhals et al, 2023** | **Intervention** | 53.38 (11.91) | 95.8 | 73.0 (10.3) | 58 |
|  | **Control** | 53.54 (14.05) | 79.2 | 74.3 (8.5) | 50 |
| * **Mean (SD)**  **# Median (IQR)** | | | | | |

**Appendix 4**

**Risk of bias results using ROB-2 Tool for RCTs.**

| **Reference** | **ROB arising from the Randomization process.** | **ROB deviations from the intended interventions** | **Missing outcome data** | **Risk of bias in measurement of the outcome** | **Risk of bias in selection of the reported result** | **Overall risk of bias** |
| --- | --- | --- | --- | --- | --- | --- |
| **Van den heuvel et al, 2002** | High | Low | Low | Some concerns | Some concerns | High |
| **Grant et al, 2002** | Low | Low | Some concerns | Low | Low | Some concerns |
| **Hartke et al, 2003** | Some concerns | High | Low | High | High | High |
| **Larson et al, 2005** | Low | Low | Low | Low | Some concerns | Some concerns |
| **Draper et al, 2007** | Low | Low | Some concerns | Low | Some concerns | Some concerns |
| **King et al, 2012** | Low | Low | Low | Low | Some concerns | Some concerns |
| **Pfeiffer et al, 2014** | Low | Low | Low | Low | Some concerns | Some concerns |
| **Ana Rodríguez-Gonzalo et al, 2015** | Low | Low | Low | Low | Some concerns | Some concerns |
| **Inci et al, 2016** | Low | Some concerns | Low | Some concerns | Some concerns | Some concerns |
| **Goudarzian et al, 2018** | Low | High | Low | Low | Low | High |
| **Hekmatpou D et al, 2019** | Low | Low | Some concerns | Low | Some concerns | Some concerns |
| **Day et al, 2020** | Low | Low | High | Low | Some concerns | High |
| **Farahani et al, 2021** | Low | Low | Some concerns | Some concerns | Some concerns | Some concerns |
| **Ardalan et al, 2022** | Low | Some concerns | Some concerns | Some concerns | Some concerns | High |
| **Elsheikh et al, 2022** | Low | Low | High | High | Some concerns | High |
| **Bierhals et al, 2023** | Low | Low | Low | Low | Low | Low |

| **Reference** | **Pre-intervention** | | **At intervention** | **Post-intervention** | | | | **Overall risk of bias** |
| --- | --- | --- | --- | --- | --- | --- | --- | --- |
|  | **Bias due to confounding** | **Bias in selection of participants into the study** | **Bias in classification of interventions** | **Bias due to deviations from intended interventions** | **Bias due to missing data** | **Bias in measurement of outcomes** | **Bias in selection of the reported result** |  |
| **Grasel et al, 2005** | Serious | Low | Low | Low | Critical | No information | Low | Critical |
| **Araujo et al, 2018** | Low | Low | No information | No information | Low | No information | Low | No information |

**Appendix 5**

**Risk of bias results using ROBINS-1 Tool for non-randomized studies of intervention.**


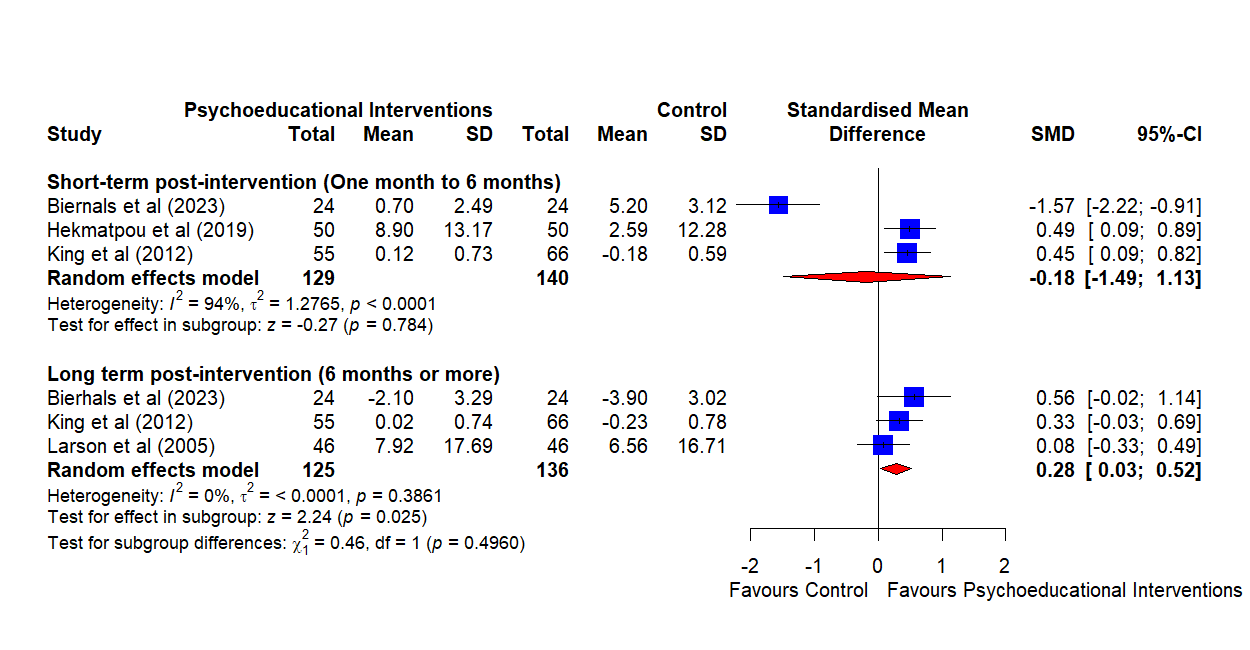


**Figure S1.** Subgroup analysis based on the duration of follow-up for quality of life outcome


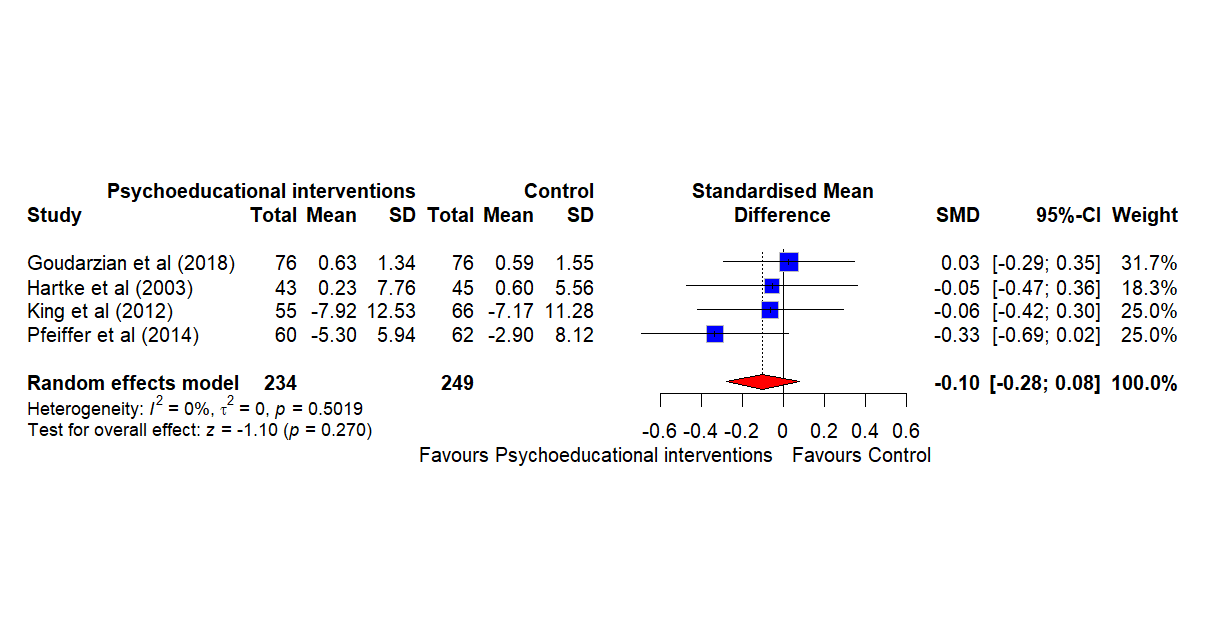


**Figure S2.** Forest plot of depression level after excluding the non randomized clinical trial, Grasel et al, 2005 [47]**.**


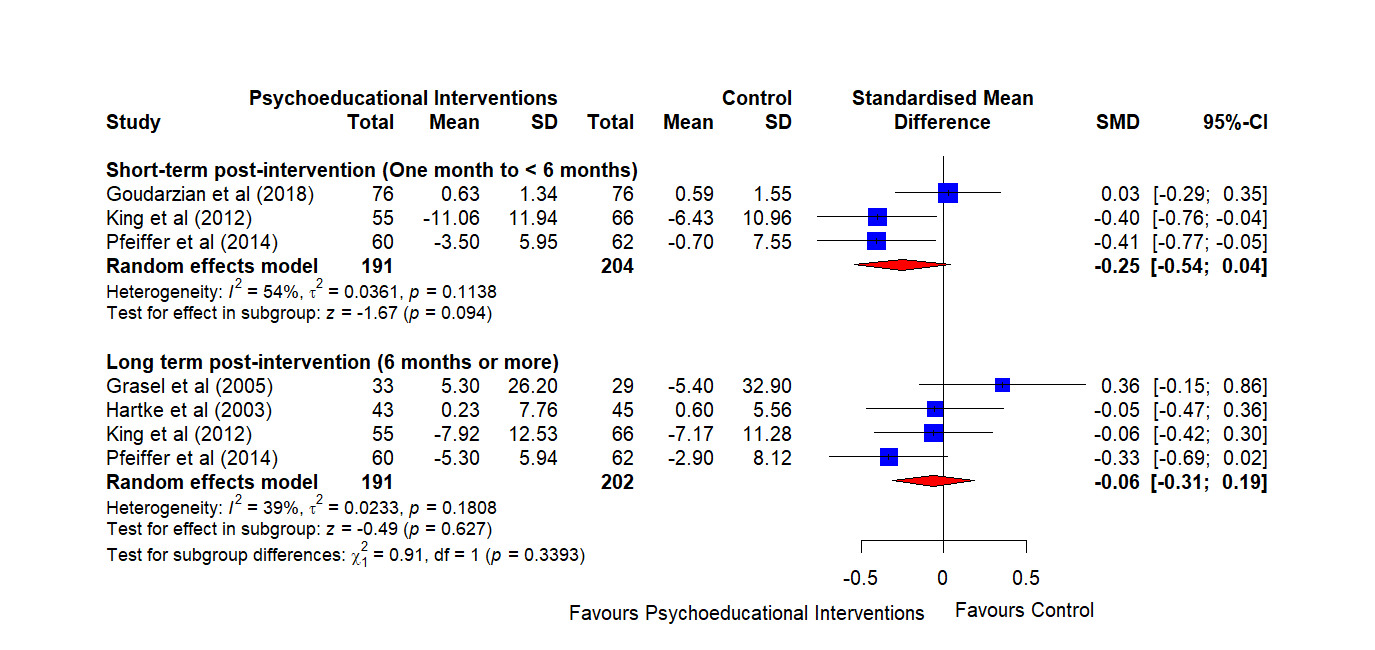


**Figure S3.** Subgroup analysis based on the duration of follow-up for depression level outcome


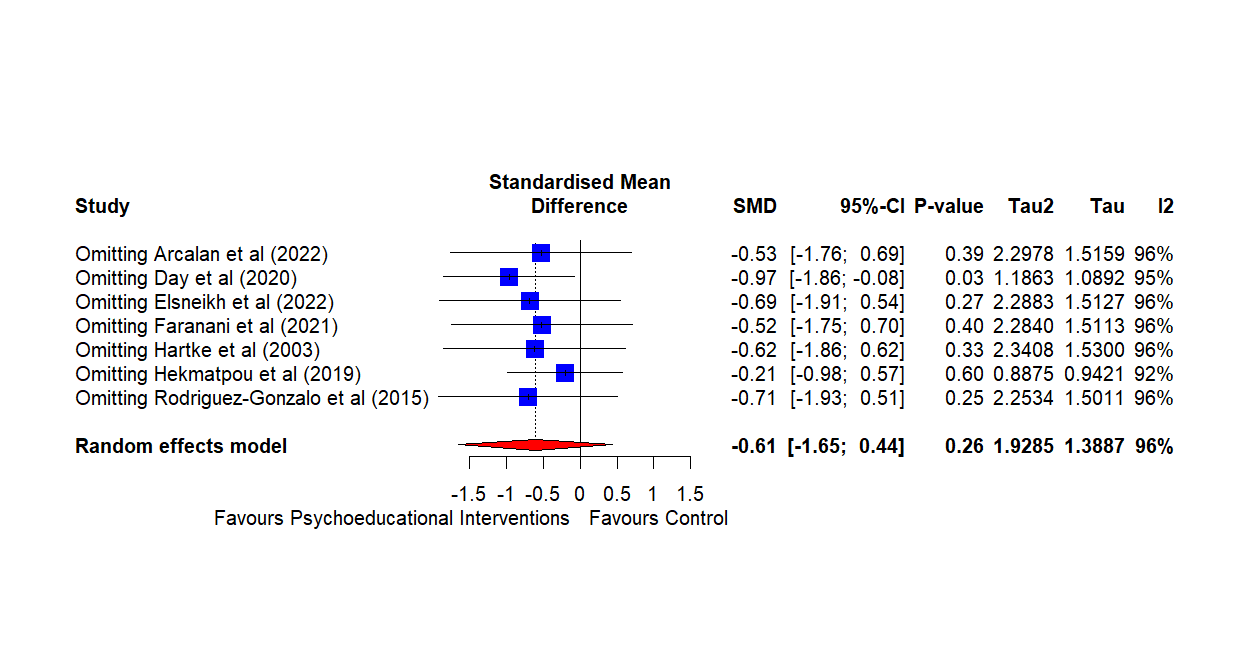


**Figure S4.** Sensitivity analysis of burden outcome.


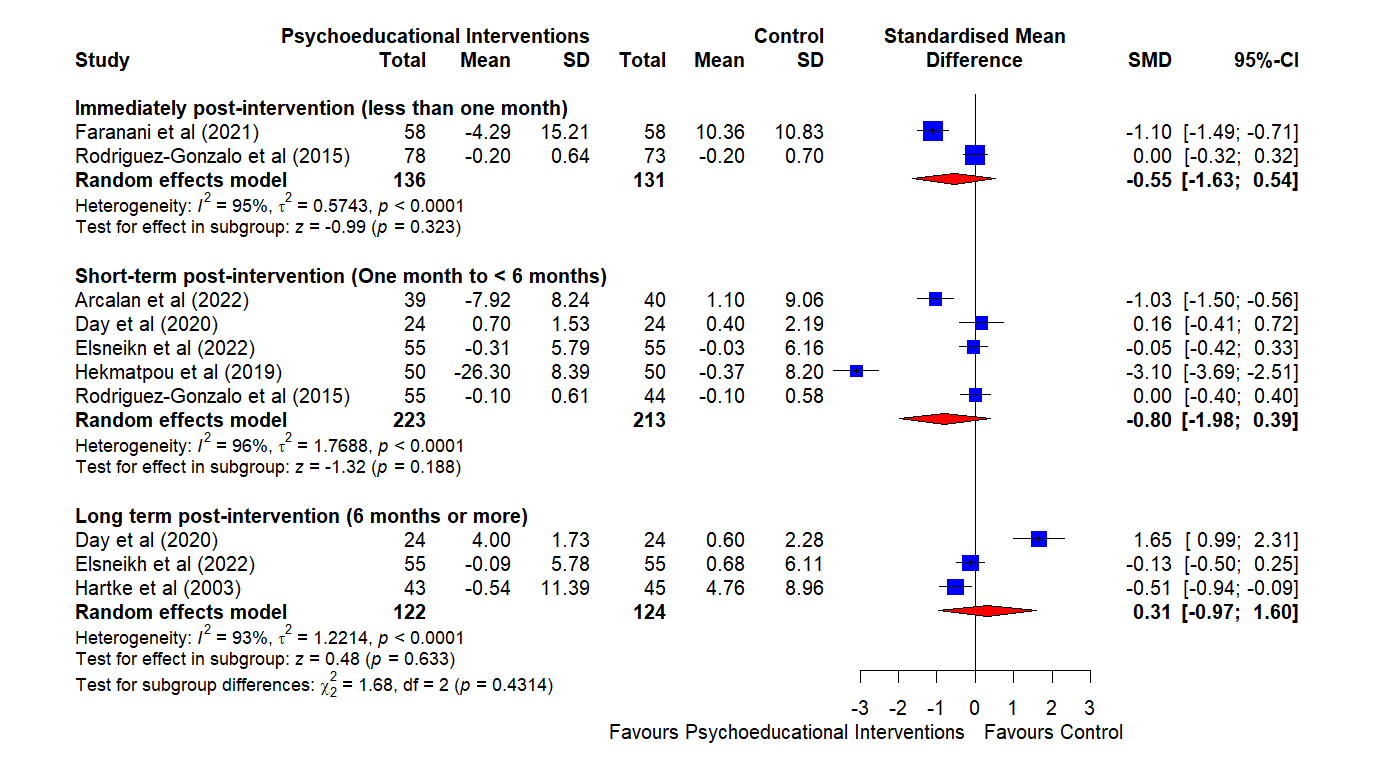


**Figure S5.** Subgroup analysis based on the duration of follow-up for burden outcome
